# UDP-glucuronosyltransferase UGT1A7 genetic polymorphisms in hepatocellular carcinoma: a differential impact according to seropositivity of HBV or HCV markers?

**DOI:** 10.1186/1471-2407-7-214

**Published:** 2007-11-19

**Authors:** I Stücker, MA Loriot, G N'Koutchou, S Cénée, L Bodin, C Mulot, M Gelu-Simeon, L Pelletier, JP Bronowicki, F Degos, P Beaune, P Laurent-Puig, D Hémon, JC Trinchet, G Pelletier

**Affiliations:** 1 Inserm, U754, Villejuif, F-94807 France; 2 UMR-S754, IFR69, Villejuif, F-94807 France; 3 Inserm U775, Paris, F-75005 France; 4 UMR-S775, Paris, F-75005 France; 5 Hôpital Jean Verdier, Bondy, F-93143 France; 6 CHU de Bicêtre, Le Kremlin Bicêtre, F-94270 France; 7 CHU de Nancy, Vandoeuvre les Nancy, F-54500 France; 8 Hôpital Beaujon, Clichy, F-92110 France

## Abstract

**Background:**

We conducted a case-control study to evaluate the role of UDP-glucuronosyltransferase 1A7 (UGT1A7) polymorphisms in the onset of hepatocellular carcinoma (HCC).

**Methods:**

The study included 165 patients with HCC, 134 with cirrhosis and 142 controls without liver disease, matched for age and hospital. All were men younger than 75 years. HCC and cirrhosis patients were stratified according to time since cirrhosis diagnosis.

**Results:**

We found a positive association between the UGT1A7*3/*3 genotype and HCC when the comparison was restricted to patients whose disease was of viral origin [OR = 3.4 (0.3–45)] but a negative association when it included only alcoholic patients [OR = 0.1 (0.02–0.6), p = 0.01].

**Conclusion:**

Our study shows that UGT1A7 may play a role in hepatocellular carcinogenesis and that this role may differ according to the primary cause of the cirrhosis.

## Background

Hepatocellular carcinoma (HCC) is a major cancer in developing countries. Etiologically it is a multifactorial disease that has been linked to both viral and chemical carcinogens. Established causal risk factors include hepatitis B (HBV) infection, dietary aflatoxin exposure, chronic alcohol consumption, and cirrhosis of the liver [1]. IARC recently listed smoking as a cause of liver cancer, although the dose-effect relation is not firmly established [2]. Hepatitis C virus (HCV) also appears to have contributed to the increasing incidence of HCC in North America and Europe over the past two decades and will probably become the dominant viral cause of this cancer in these low-risk regions. In France, around 90% of HCC occur on cirrhotic livers, with heavy drinking the principal causal factor.

Accumulating evidence indicates that susceptibility to cancer is mediated by genetically determined differences in the effectiveness of carcinogen detoxification. Various epidemiologic studies have examined the role in HCC of different polymorphisms [3], including UDP-glucuronosyltransferase 1A7 (UGT1A7) [4-6].

The human UDP-glucuronosyltransferases (UGTs) are an enzyme superfamily that metabolizes endogenous compounds such as bilirubin, steroid hormones and environmental carcinogens including tobacco-specific nitrosamines and benzo(a)pyrene by glucuronidation reaction [7]. Nine alleles of UGT1A7 have been described: alleles *3 and *4 are associated with decreased enzyme activity and allele *2 with an activity similar to the wild-type allele *1 [7]. The relation between the UGT1A7 polymorphism and HCC was first investigated by Vogel et al in a German population [4]. In that study, subjects carrying one allele with a low detoxification capacity (UGT1A7*3) were at higher risk of HCC. Two subsequent studies have confirmed this result in different populations, one Japanese and the other Taiwanese [5,6]. Other epidemiological studies of different cancer sites also suggest that low UGT1A7 detoxification activity is associated with a higher risk of cancer [8-12]; one study was unable to confirm this result [13].

The case-control study reported here sought to evaluate the role of UGT1A7 polymorphisms in the onset of HCC. Since almost all HCC cases occur in cirrhotic liver, we considered 2 control groups, one with no liver disease and one with cirrhosis and no liver cancer.

## Methods

The study took place in the hepatology departments of 4 hospitals, 3 in the Paris area and 1 in eastern France (Nancy). Subjects (HCC cases, cirrhosis patients and controls) were recruited prospectively from March 2000 to August 2003.

### HCC cases

Eligible cases were patients aged 75 years or younger, born in Europe of parents born in Europe, admitted to one of the participating departments and newly diagnosed with primary HCC, on the basis of either histologic analysis or the combination of focal lesions detected by any imaging technique and an alpha feto-protein (AFP) level > 250 ng/ml. Of the 220 eligible HCC patients identified, 165 (75%) were interviewed (20 refused to participate, 7 died and 28 were lost to follow-up before they could be included). Of those interviewed, 151 (91.5%) had serum samples tested for hepatitis B surface antigen (HbsAg) and antibodies to HCV (anti-HCV).

### Cirrhosis patients

Patients with cirrhosis but not HCC were recruited in the same departments as the cancer patients. Cirrhosis was defined either by histology or by the combination of clinical, laboratory, and endoscopic signs. The absence of HCC was established by the absence of focal lesions on imaging and by an AFP level < 10 ng/ml. Cirrhosis patients were stratified in 3 classes according to time since cirrhosis diagnosis: i) subjects with newly-detected cirrhosis were matched for age (± 5 years) with cases whose cirrhosis was diagnosed at the same time as the carcinoma, ii) subjects with cirrhosis diagnosed within the past 5 years, matched for age with HCC cases whose cirrhosis had been diagnosed for more than 1 and fewer than 5 years, and iii) subjects with cirrhosis diagnosed for more than 5 years, matched for age with HCC cases whose cirrhosis had also been diagnosed more than 5 years earlier.

We preferred complying with this stratification criterion, even if age matching then failed, because we thought that it was most important to avoid comparing new cirrhosis patients with HCC patients whose cirrhosis had begun long ago.

We tested serum samples of the cirrhosis patients for the presence of hepatitis B surface antigen (HbsAg) and antibodies to HCV (anti-HCV), as we had for the HCC patients.

### Controls

Controls without liver disease were recruited in different hospital departments at the same time as the cases. Each time a new HCC patient was included in the study, we sought a control subject who met the matching criteria (age ± 2.5 years and hospital) and the additional inclusion criteria: no history of cancer and well enough to provide a blood sample. We systematically took the first person who met these criteria. Nearly all agreed to participate; when they did not, we took the next eligible control who did. This control group comprised patients with a total of 51 different diagnoses including coloscopy (17%), diseases of the circulatory system (40%), diseases of the digestive system (11%), diseases of the musculoskeletal system and connective tissue (10%), and finally diverse diseases, each representing less than 10%. The mean number of patients per diagnosis was 3 (min = 1 max = 22 admitted for coloscopy).

Written informed consent was obtained from all subjects and study approval was granted by the institutional ethics committee

### Questionnaire data

All HCC patients, cirrhosis patients, and controls were interviewed face-to-face in the hospital, according to a questionnaire that asked for information about social and demographic characteristics and then for specific information about lifetime tobacco use. A food frequency questionnaire completed the interview. It included questions about beverages, alcoholic and nonalcoholic. Subjects were asked about the frequency of their consumption of beer, wine, and spirits. On the assumption that a glass of beer (250 ml), a glass of wine (120 ml), a "strong" spirit (i.e., whisky) (4 ml), and a "lighter" spirit (i.e., port) (12 ml) had respective ethanol contents of 8.75 g, 9.6 g, 9.38 g, and 6.08 g, we converted alcoholic beverage consumption into grams of pure ethanol to obtain a cumulative lifetime ethanol dose and then divided by the total duration of alcoholic beverage consumption for a mean weekly consumption, which we expressed as drinks per day. Because the food frequency questionnaire was not available at the beginning of the study, the first 50 subjects did not complete it, and the alcohol variable is missing for them.

### DNA bank and genotyping

#### DNA extraction

Genomic DNA was purified from human lymphocytes (HCC cases and cirrhosis patients and controls) with a commercial kit (Qiagen, Courtabœuf, France) and stored at -20°C until use.

DNA (10–50 ng/μl) was used for polymerase chain reaction (PCR). All PCRs were performed in a 25-μl reaction volume containing 2 μl of DNA, 2.5 μl of 10× PCR buffer (*GeneAmp*, Applied Biosystems, Courtabœuf, France), MgCl_2 _(*GeneAmp*, Applied Biosystems) at various final concentrations, 200 μM dNTPs (ABgene, Courtabœuf, France), 400 nM of each primer (Genset, Paris, France) and Taq Polymerase (*AmpliTaq *DNA Polymerase, Applied Biosystems). PCR was performed in a DNA thermal cycler (Applied Biosystems). The amplified products (5 μl) were electrophoresed in 1.5% agarose gel (Invitrogen, Cergy Pontoise, France) and visualized by ethidium bromide staining.

The UGT1A7 protein sequences differ at amino acid positions 129, 131 and 208. The various combinations create four distinct allelic variants in human populations: UGT1A7*1 (N129R131W208), *2 (K129K131W208), *3 (K129K131R208) and *4 (N129R131R208). Haplotype analysis revealed that the polymorphisms at position 129 and 131 are in complete disequilibrium linkage, whereas the polymorphism at position 208 occurs independently [14]. Based on these data, two polymorphisms (e.g., N129K and W208R) were detected for the *UGT1A7 *genotyping.

The single nucleotide polymorphisms (SNPs) of *UGT1A7 *(N129K and W208R) were detected by using a 5' nuclease allelic discrimination assay (ABI PRISM 7700 Sequence Detection System; Applied Biosystems, Foster City, CA). The following sequences were used for the primers for amplification of PCR fragments containing SNPs of the *UGT1A7 *gene and for specific probes (for allelic discrimination):

N129K Forward: 5'-CACCATTGCGAAGTGCATTT-3'

(AA**T**->AA**G**) Reverse 5'-GGATCGAGAAACACTGCATCAA-3'

Probe N129 5'-CAGGAGTTTGTTTAA**T**GAC-3'

Probe K129 5'-CAGGAGTTTGTTTAA**G**GAC-3'

W208R Forward: 5'-CCAGACTTCTCTTAGGGTTCTCAGA-3'

(**T**GG->**C**GG) Reverse 5'-CAGAGGCTATTTCTAAGACATTTTTGA-3'

Probe W208 5'-CATGATGTGGTTCC**A**TAC-3'

Probe R208 5'-CATGATGTGGTTCC**G**TAC-3'

Specific probes for each allele were labeled with the fluorescence reporter dyes FAM and VIC at their 5' extremities.

We classified these alleles into 3 groups on the basis of their enzymatic activity *1/*1 or *1/*2 or *2/*2: high activity, *1/*3 or *2/*3 intermediate activity, *3/*3: low activity [7].

### Statistical Method

All statistical analyses were performed using STATA software. Odds ratios (OR) and their 95% confidence intervals (95% CI) were calculated with unconditional logistic regression including the matching variables of age and hospital as well as the standard risk factors (such as drinks/day) for the comparison between cases and controls and time since cirrhosis diagnosis (in years), cirrhosis characteristics (heavy drinkers-virus(-), virus(+)), and social class (blue- or white-collar workers) for the comparison between the HCC cases and the cirrhosis controls.

The role of UGT1A7 was tested for each genotype, with the genotype *1/*1 as the reference category, and then for each phenotype, with high enzyme detoxification activity as the reference category. The phenotypic classification that we have adopted pools the *1/*1 or *1/*2 or *2/*2 to define subjects with a high activity, those with genotypes *1/*3 or *2/*3 to define subjects with intermediate activity, while genotype *3/*3 defines low activity [7].

Interactions for a multiplicative effect between genes or between environmental factors and genetic polymorphisms were tested with a case-only approach. The interaction OR with a case-only approach is noted as prevalence odds ratio [15,16]. This approach requires verification that the distribution of the two factors (i.e. genetic and environmental) is independent among controls.

All reported values are 2-tailed.

## Results

Table 1 presents the main characteristics of the subjects included in the survey. The mean age of HCC cases was 62 years. Half were classified as blue-collar workers according to the job title of their last job. Twenty-nine (21%) had had a cirrhosis diagnosis for more than 5 years when HCC was diagnosed, 42 (31%) for 1 to 4 years and 65 (48%) simultaneously to or during the same year as the HCC diagnosis. Serum samples of 151 HCC patients were tested for virus markers, and 40 were positive. Seventeen of these 40 were also heavy drinkers, while there were 102 heavy drinkers who were negative for virus markers (62%). We therefore sub-classified the HCC cases in two groups, those who were negative for virus markers — all of whom were heavy drinkers (heavy drinker-Virus(-)) — and those positive for virus markers, 42% of whom were also heavy drinkers (Virus(+)).

**Table 1:** Description of the population

	Controls		Cirrhosis		HCC		HCC vs Controls		HCC vs Cirrhosis	
	N	%	N	%	N	%	OR	95%CI	OR	95%CI
**Age**										
< 50	11	7.7%	22	16.4%	17	10.3%	1.0	Ref	1.0	Ref
50–60	39	27.5%	63	47.0%	46	27.9%	0.8	0.3–1.9	1.0	0.5–2.1
60–70	56	39.4%	32	23.9%	61	37.0%	0.7	0.3–1.6	2.9	1.3–6.3
> 70	36	25.4%	17	12.7%	41	24.8%	0.7	0.3–1.8	3.8	1.6–9.3
	142		134		165					
m ± sd	62 ± 8.5		58 ± 8.6		62 ± 8.9					
										
**Hospital**										
K.-Bicêtre	53	37.3%	45	33.6%	48	29.1%				
Bondy	48	34.0%	53	39.1%	60	36.4%	-	-	-	-
Nancy	13	9.0%	16	12.0%	25	15.1%	-	-	-	-
Beaujon	28	20.0%	20	15.0%	32	19.4%	-	-	-	-
	142		134		165					
**Social class**										
White-collar worker	79	56.0%	49	38.0%	82	50.0%	1.0	Ref	1.0	Ref
Blue-collar worker	63	44.0%	80	62.0%	82	50.0%	1.3	0.8–2.0	0.6	0.3–0.9
	142		129		164					
**Time since cirrhosis diagnosis**										
0–1 yr			53	43.4%	65	47.8%	-	-	1.0	Ref
> 1–< 5			43	35.2%	42	30.9%	-	-	0.8	0.4–1.5
> 5			26	21.3%	29	21.3%	-	-	0.8	0.4–1.5
			122		136					
**Cirrhosis characteristics**										
Heavy drinkers-virus(-)			106	79.1%	102	61.8%				
Virus(+) not heavy drinkers			10^1^	7.5%	23^2^	13.9%				
Heavy drinkers-virus(+)			10^3^	7.5%	17^4^	10.3%				
Hemochromatosis			1	0.7%	6	3.6%				
other or unknown			7	5.2%	17	10.3%				
					165					

**1**: 6 anti-HCV (+) 3 HbsAg (+) 1 both; **2**: 20 anti-HCV (+) 2 HbsAg (+) 1 both ; **3**:9 anti-HCV (+) 1 HbsAg (+); **4**:15 anti-HCV (+) 2 HbsAg (+);

Control patients with cirrhosis were slightly younger than HCC cases (m = 58 ± 9 years), and 82 (62%) were blue-collar workers. Distribution of time since cirrhosis diagnosis was similar to that of HCC cases with 21%, 35% and 43% of the cirrhosis patients diagnosed respectively for more than 5 years, 1 to 4 years and during the year of the interview. Cirrhosis was attributed to chronic alcohol use for 106 patients negative for virus markers (79%). Twenty subjects had serum samples positive for viral markers (HBV or HCV or both), 10 of whom were also heavy drinkers. We sub-classified the cirrhosis patients into the same two groups as the HCC cases: those who had negative virus markers, all of whom were heavy drinkers (heavy drinker-Virus(-)), and those who had positive virus markers, half of whom were heavy drinkers (Virus(+)).

Controls with no liver disease were the same age as HCC cases (m = 62 ± 8.5), and 63 (44%) were classified as blue-collar workers.

### Relations between HCC and known risk factors (alcohol and hepatitis)

Table 2 reports the distribution of controls, cirrhosis patients and HCC cases according to their alcohol consumption and includes the related OR estimates. Comparing the HCC and control subjects shows that the OR increased strongly with alcohol consumption. We estimated an OR of 3.7 per unit of 10 drinks/day (p < 10^-3^). The comparison between HCC and cirrhosis patients showed no association with alcohol consumption, and the OR per unit of 10 drinks per day was 1.07 (p = 0.7).

**Table 2:** Classic risk factors of HCC: Alcohol consumption and HBV/HBC

	Controls (N = 142)		Cirrhosis (N = 134)		HCC (N = 165)		HCC vs Controls		HCC vs Cirrhosis	
	N	%	N	%	N	%	OR^a^	95%CI	OR^b^	95%CI
**Drinks/day**^c^										
0–3	58	44,3%	17	14,0%	23	17,2%	1.0	Ref	1.0	Ref
3–7	38	29,0%	23	19,0%	26	19,4%	1.8	0.9–3.7	1.4	0.5–4.1
7–11	21	16,0%	25	20,7%	40	29,9%	4.9	2.4–10.2	2.7	0.9–7.5
≥ 11	14	10,7%	56	46,3%	45	33,6%	9.5	4.2–21.1	1.5	0.6–4.0
	131^c^	100,0%	121^c^	100,0%	134^c^	100,0%				
m ± sd	5.2^d ^± 5.2		11.2^d ^± 8.1		9.7^d ^± 9.4		3.7^f^	2.1–6.4	1.07^f^	0.97–1.5
m ± sd	3.3^e ^± 3.4		6.7^e ^± 8.3		5.1^e ^± 7.1					
										
**Cirrhosis characteristic**										
Heavy drinkers-virus(-)			106	84,1%	102	71,8%			1.0	Ref
Virus(+)			20	15,9%	40	28,2%			3.1	1.5–6.3

**a: **adjusted for age and hospital **b: **adjusted for age, hospital, time since cirrhosis diagnosis, origin of the cirrhosis (viral(+) or not), and social class **c**: alcohol variable missing for 50 subjects because the food frequency questionnaire was not available at the beginning of the study **d**: Lifetime mean consumption **e: **Consumption at the time of the interview. **f: **OR per unit of 10 drinks/day

We also compared the prevalence of HbsAg and HCV antibodies among subjects with HCC and those with cirrhosis only and found, as expected, a higher prevalence of virus-positive subjects among the subjects with HCC [OR = 3.1 (1.5–6.3)], with alcohol-induced cirrhosis as the reference group.

### Polymorphisms of xenobiotic metabolic enzymes

Table 3 presents the results of the UGT1A7 genotyping. The alleles at the individual loci were in Hardy-Weinberg equilibrium in all three groups.

**Table 3:** Association between UGT1A7 and HCC

		Controls		Cirrhosis		HCC		HCC vs Controls		HCC vs Cirrhosis	
		N	%	N	%	N	%	OR^1^	95% CI	OR^2^	95% CI
All											
	*1/*1	12	9.5%	11	8.2%	28	17.0%	1.0	Ref	1.0	Ref
	*1/*2	27	20.6%	23	17.2%	31	18.8%	0.5	0.2–1.3	0.8	0.3–2.5
	*1/*3	36	27.5%	36	26.9%	51	30.9%	0.8	0.3–2.0	0.8	0.3–2.3
	*2/*2	9	6.9%	10	7.5%	12	7.3%	0.6	0.2–2.3	0.8	0.2–3.3
	*2/*3	28	21.4%	31	23.1%	19	11.5%	0.4	0.1–1.2	0.3	0.1–1.2
	*3/*3	19	14.5%	22	16.4%	23	13.9%	0.7	0.2–2.1	0.4	0.1–1.2
	determined	131		133		164					
											
	H	48	33.8%	44	32.8%	71	43.0%	1.0	Ref	1.0	Ref
	HL	64	45.1%	67	50.0%	70	42.4%	1.0	0.5–1.8	0.7	0.4–1.4
	L	19	13.4%	22	16.4%	23	13.9%	1.2	0.5–2.6	0.4	0.2–1.0
		131		133		164					

Heavy drinkers virus(-)											
	*1/*1			7	6.5%	18	17.5%	1	Ref	1	Ref
	*1/*2			17	15.9%	23	22.3%	0.7	0.2–2.3	0.7	0.2–3.0
	*1/*3			29	27.1%	31	30.1%	1.1	0.4–3.2	0.5	0.1–2.0
	*2/*2			10	9.3%	9	8.8%	1.1	0.3–4.4	0.5	0.1–2.4
	*2/*3			27	25.2%	12	11.6%	0.4	0.1–1.5	0.2	0.04–0.7
	*3/*3			17	15.9%	10	9.7%	0.5	0.2–1.9	0.1	0.02–0.6
	determined			107		103					
											
	H			34	31.8%	50	48.5%	1.0	Ref	1.0	Ref
	HL			56	52.3%	43	41.7%	0.6	0.3–1.1	0.5	0.2–1.1
	L			17	15.9%	10	9.7%	0.5	0.2–1.2	0.2	0.04–0.6
				107		103					

Virus(+)											
	*1/*1			3	15.0%	6	15.0%	1.0	Ref	1.0	Ref
	*1/*2			4	20.0%	6	15.0%	0.6	0.1–3.9	1.3	0.1–19.7
	*1/*3			6	30.0%	11	27.5%	1.3	0.2–7.0	1.8	0.1–19.2
	*2/*2			0	0.0%	3	7.5%	0.9	0.1–8.0	-	
	*2/*3			3	15.0%	4	10.0%	0.9	0.1–5.8	6.4	0.3–140
	*3/*3			4	20.0%	10	25.0%	2.3	0.4–13.3	3.4	0.3–45
	determined			20		40					
											
	H			7	35.0%	15	37.5%	1.0	Ref	1.0	Ref
	HL			9	45.0%	15	37.5%	1.5	0.6–4.1	1.6	0.2–8.3
	L			4	20.0%	10	25.0%	3.1	1.0–9.6	2.2	0.3–15.3
				20		40					

1: adjusted for age, hospital, drinks/day, social class.; 2: adjusted for age, hospital, drinks/day, social class, time since cirrhosis diagnosis

Compared with the controls, HCC was not significantly related to any of the different genotypes and all ORs were less than 1. The negative ORs were not significant. Analysis of the enzyme activity phenotype also showed no particular association between UGT1A7 polymorphism and HCC. Distribution of the genotypes was similar for cirrhosis patients and controls. Accordingly, our comparison of the genotype distribution in HCC and cirrhosis patients again showed ORs less than 1 and not significant. Analysis according to enzymatic activity showed an OR of 0.4 [0.1–1.1] for subjects defined with low activity (with high activity as the reference).

Interestingly, the association between UGT1A7 and HCC risk seemed to differ according to characteristics of cirrhosis. The initial comparison between HCC cases and controls was restricted to HCC cases who were heavy drinkers and virus(-). The relation with UGT1A7 was less than 1 and not significant, especially for subjects with low enzymatic activity [OR = 0.5 (0.2–1.2)]. But when we considered HCC cases who were virus(+), the OR of HCC for the UGT1A7*3/*3 genotype was 2.3 (0.4–13.3). This pattern of a rather negative OR among patients who were heavy drinkers and virus(-) and a non-significant positive OR among patients who were virus(+) was clearly reinforced when we compared the HCC cases with the cirrhosis patients. Among virus(+) subjects, the OR of HCC for the UGT1A7*3/*3 genotype was 3.4 (0.3–45) and 2.2 (0.3–15) when we classified the subjects according to enzymatic activity and 0.1 (0.02–0.6) (p = 0.01) and 0.2 (0.04–0.6) (p = 0,007) respectively, for virus(-) heavy-drinking subjects. We estimated this interaction with a case-only approach. We first verified that there was no association between the two factors (presence of a viral marker and UGT1A7 polymorphism — either genotypic or phenotypic) among cirrhosis patients: the association for the genotypic form of the UGT1A7 polymorphism had a X^2 ^= 4.7, p = 0.45 and the phenotypic form, X^2 ^= 0.38, p = 0.82. The prevalence odds ratio for virus(+) and UGT1A7 enzymatic activity was 2.3 (0.8–6.4) for the intermediate activity category and 15.1 (2.7–84) (p = 0.002) for the low activity category. The same analysis with the genotype data showed a prevalence odds ratio for virus(+) and genetic polymorphism of 14.4 (2.0–103) (p = 0.008) for the UGT1A7*3/*3 genotype.

Finally, we conducted an analysis that separated the virus(+) subgroup into those who were not heavy drinkers (i.e. 23 cases and 10 cirrhosis cf table 1) and those who were. The results, despite the relatively small number of subjects, show an OR of HCC associated with the genotype UGT1A7*3/*3 of 5.3 [0.2–153], but of 0.5 [0.02–12] among those virus(+) subjects who were heavy drinkers.

## Discussion

The aim of our study was to assess whether the UGT1A7 polymorphism might increase the risk of HCC. We considered 2 separate control groups. The first comprised controls without liver disease to investigate whether these genetic susceptibility factors were risk factors for hepatic carcinoma. In addition, since almost all HCC in France occurs on cirrhotic liver, we also considered a control group of patients with cirrhosis to investigate whether these polymorphisms were risk factors for the transformation from cirrhosis to carcinoma.

The study we set up used a hospital-based approach. our controls were selected from more than 13 departments to avoid overrepresentation of a single disease that might be related to the environmental or genetic risk factors we were studying. We also included a second control group of cirrhosis patients without HCC. Most cases of HCC (approximately 90%) develop in cirrhotic liver, and time is the principal risk factor: roughly 3–4% of cirrhosis patients per year develop HCC. It was thus essential to avoid comparing patients with newly diagnosed cirrhosis, that is, who had not had enough time to develop HCC, to HCC patients with cirrhosis diagnosed several years earlier. We therefore stratified HCC and cirrhosis patients by time since cirrhosis diagnosis and included newly diagnosed cirrhosis patients as controls only for HCC patients whose cirrhosis was diagnosed at the same time as the carcinoma (stratum 1); cirrhosis patients diagnosed within the past 5 years served as controls for HCC patients whose cirrhoses were also diagnosed in that period (stratum 2), and finally cirrhosis patients diagnosed more than 5 years earlier (stratum 3) as controls for HCC patients whose cirrhoses were also diagnosed more than 5 years previously.

We evaluated subjects' history of alcohol consumption except for the first 50 subjects, because the food frequency questionnaire was not available at the time we started the study. These data are therefore missing totally at random and it is very likely that it did not create any selection bias in the analysis of the different risk factors, particularly genetic [17].

The results for UGT1A7 appear to be related to the characteristics of cirrhosis. The genotypic frequencies that we observed among our controls are very similar to those reported by Guillemette et al. in a population of normal healthy blood donors from the USA [14]. The alleles at the individual loci were in Hardy-Weinberg equilibrium in all three groups. In our comparisons between cases and controls, we observed a non-significant positive OR with UGT1A7*3/*3 when we restricted the analysis to virus(+) subjects [OR = 2.3 (0.4–13.3)]. We obtained similar results when we compared the virus(+) cases and the cirrhosis-viral(+) group [OR = 3.4 (0.3–45)]. These results are consistent with Vogel's study, which reported a strong association with the UGT1A7*3/*3 genotype [4]. We note that the HCC patients in that study had mainly viral disease. A more recent case-control study in a population of patients with viral HCC also found a relation between UGT1A7 low enzymatic activity and HCC; its results are quantitatively very similar to ours [2.0 (0.6–6.7) for the UGT1A7*3/*3 genotype and 2.7 (1.4–5.3) for the low enzymatic activity [5]. This result was again reproduced in a third case-control study in a Taiwanese population where HBV and HCV infections are endemic [6]. Inversely, we found a strong negative OR for this genotype (UGT1A7*3/*3) when we considered HCC and cirrhosis patients who were heavy drinkers and virus(-). Furthermore, our results show a strong positive association with the UGT1A7*3/*3 genotype for those with viral markers who were not heavy drinkers [OR = 5.3 (0.2–153)], a negative association among heavy drinkers with viral markers [OR = 0.5 (0.02–12)], and finally a strong negative association for heavy drinkers who were virus (-) [OR = 0.1 (0.02–0.6), p = 0.001]. These results seem extremely interesting, despite the large confidence intervals.

Our case control study included a population of HCC and cirrhosis patients, most of whom were heavy drinkers and virus(-) (i.e. 79% of HCC patients and 62% of cirrhosis patients). The distribution of the characteristics of the cirrhosis (i.e. virus(+) or heavy drinker-virus(-)) of our cases and cirrhosis patients is markedly different from those found in the three case control studies thus far published [4-6]. Moreover the cirrhosis group was stratified according to time since diagnosis so that we did not compare HCC patients with cirrhosis patients who would not have had time to develop a carcinoma.

Because HBV/HCV viral markers were not available for controls, we used a case-only approach to assess the hypothesis that the two categories of cases, distinguished by the presence or the absence of the viral marker, were characterized by etiological heterogeneity. Our result was very strong [OR = 12.0 (1.6–92)], although its confidence interval was very wide, when we tested interaction for the *3/*3 genotype. Similarly, the prevalence OR associated with the presence of viral markers was 15.2 (2.7–86) when we tested interaction with phenotypic activity of UGT1A7. Thus, despite the low p values, the observed interaction may be due to chance. However, if true, this interaction would imply that viral induced liver carcinogenesis is favoured by the level of detoxifying UDPGT.

Functional characterization shows that the UGT1A7*3 allele has the lowest relative activity and UGT1A7*1 and UGT1A7*2 the highest [7]. We have however no hypothesis to explain the difference in the relation between HCC and UGT1A7 according to cause of cirrhosis. The UGT1A7 polymorphism appears to be related to risks for other cancer sites, including the colon, pancreas, UADT, and mouth and larynx [8-13]. There is no known viral etiology for any of these cancer sites. Further studies are needed to confirm these results and to explain why the same genotype (i.e., UGT1A7*3/*3) is more frequent in viral HCC patients and less frequent in alcohol-induced HCC patients compared with their cause-matched cirrhotic patients.

## Competing interests

The author(s) declare that they have no competing interests.

## Authors' contributions

IS conceived of the study, and participated in its design and coordination, and wrote the manuscript; SC coordinated the collection of the data, performed the statistical analysis; MAL, LB, LP, MGM carried out the molecular genetic studies; CM coordinated the collection of the blood samples; GN, JPB, JCT, FG participated in the design of the study, coordinated the inclusion of patient in their respective departments; PB, PLP participated in the design of the study, choice of the candidat genes ; DH participated in the design of the study; GP Conceived of the study, participated in the design of the study, coordinated the inclusion of patient;

All authors have read and approved the final manuscript.

## Pre-publication history

The pre-publication history for this paper can be accessed here:


